# Dry synovitis, a rare entity distinct from juvenile idiopathic arthritis

**DOI:** 10.1186/s12969-023-00789-9

**Published:** 2023-01-23

**Authors:** Lien De Somer, Brigitte Bader-Meunier, Sylvain Breton, Sara Brachi, Carine Wouters, Francesco Zulian

**Affiliations:** 1 Department of Pediatric Rheumatology, University Hospitals Leuven, Leuven, Belgium; 2 Department of Pediatric Hematology-Immunology and Rheumatology, Necker-Enfants Malades Hospital, AP-HP, Paris, France; 3 Department of Pediatric Radiology, Necker-Enfants Malades University Hospital, Paris, France; 4 Pediatric Rheumatology Unit, Department of Woman and Child Health, University of Padua, Padua, Italy

**Keywords:** Dry synovitis, Juvenile idiopathic arthritis, Joint contracture, MRI imaging, Differential diagnosis

## Abstract

**Background:**

Dry synovitis (DS) is a rare entity as only a few cases have been reported to date. We describe the clinical features, radiological manifestations and course of DS in comparison with rheumatoid factor negative polyarticular juvenile idiopathic arthritis (RFneg-polyJIA).

**Methods:**

We performed a multicenter retrospective collection of data of DS patients who presented with progressive joint limitations without palpable synovitis, absence of elevated acute phase reactants, negative ANA and RF, and imaging showing joint and/or osteochondral involvement. For comparative purposes, we included a cohort of RF neg-polyJIA patients.

**Results:**

Twelve DS patients, 8F/4 M, with mean age at onset of 6.1 years, were included. Presenting signs comprised delayed motor development, functional limitations and/or progressive stiffness. Clinical examination showed symmetric polyarticular involvement with variable muscular atrophy. MRI showed mild, diffuse synovial involvement, without effusion. With time, signs of progressive osteochondral damage became evident, despite treatment. All patients were treated with low-dose corticosteroids and methotrexate. Anti-TNF agents were prescribed in five. The response was variable with limited joint mobility in 11/12, and need of joint replacement in 2. In comparison with a cohort of RFneg-polyJIA, DS patients presented higher number of joint involved (*p* = 0.0001) and contractures (*p* = 0.0001), less swelling (*p* = 0.0001) and prolonged diagnostic delay (*p* = 0.0001).

**Conclusion:**

DS represents a unique juvenile-onset arthropathy, distinct from polyarticular JIA. Awareness among pediatricians is essential for early recognition and proper treatment. Further studies, including synovial pathology, immunology and genetics may contribute to a better understanding of this rare disorder of childhood.

## Background

Dry synovitis (DS) is a very rare form of arthritis in childhood, presenting in an indolent way and often following a destructive course. Up to now, although not included into the current classification, it has been considered a subtype of juvenile idiopathic arthritis (JIA), an umbrella term covering different forms of chronic childhood arthritis of unknown cause [1, 2].

In 1974, Levinson gave the first detailed description of DS as a little overt synovitis, associated with a gradual development of limitation of movement and appearance of deformities, absence of systemic features and normal or modestly elevated acute phase reactants (erythrocyte sedimentation rate (ESR) and C-reactive protein (CRP)) and negative antinuclear antibodies (ANA) [3]. Since its first description, only a few case reports mentioned this entity and always within the spectrum of JIA [4–7].

Herein, we present the results of a retrospective multicenter multinational study aiming to better characterize the clinical and radiological features of DS in comparison to RF negative polyarticular JIA (RFneg-polyJIA).

## Materials and Methods

This retrospective, multicenter study included patients with DS who were followed between 2007 and 2017 at three academic Pediatric Rheumatology Centers. In agreement with the few cases published so far [4–7], the selected inclusion criteria were age at onset ≤ 16 years, presence of progressive joint limitations without palpable synovitis, imaging showing synovial, articular and/or osteochondral involvement, absence or moderately increased acute phase reactants (ESR, CRP) and negative ANA and Rheumatoid Factor (RF).

The clinical characteristics and laboratory investigations of the patients were recorded. Clinical evaluation included general systems and growth assessment, a comprehensive joint and neurological examination. ESR and CRP were considered abnormal if greater than 20 mm/hour and 5 mg/l, respectively. The differential diagnosis included genetic conditions such as neuromuscular diseases, bone dysplasias or metabolic disorders.

Imaging comprised standard radiographies, MRI with T1-T2 weighted images with or without gadolinium enhancement. An expert radiologist (SyB) reviewed all imaging data. For comparative purposes, data of a cohort of consecutive patients with RF-negative polyarticular JIA (RFneg-polyJIA), according to ILAR criteria [2], were included.

### Statistical analysis

Demographic variables and clinical features were analysed by descriptive statistics. Chi square test and Fisher's exact test were used, where appropriate, to compare the categorical variables between DS and RFneg-polyJIA. All statistical tests were 2-sided and P-values less than 0.05 were considered significant. All analyses were carried out using the SPSS statistical software (Vers. 18.0) for Windows (SPSS Inc., Chicago, IL).

The study was performed in accordance with the Declaration of Helsinki. Patients consented to participation in the study and publication of data.

## Results

**Fig. 1:**
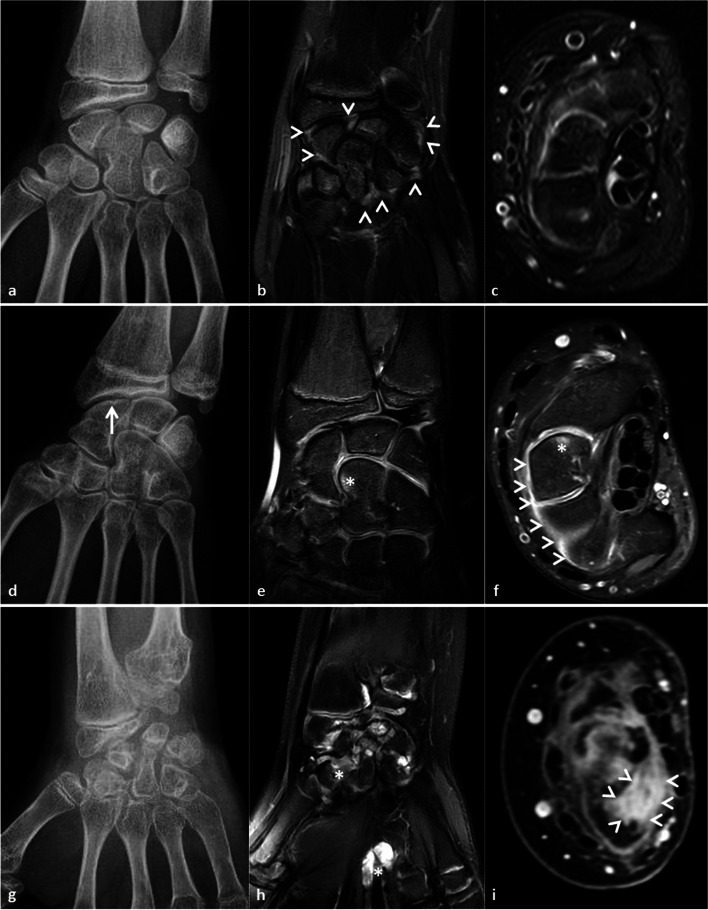
Radiological findings of Dry Synovitis in comparison with RF negative polyarticular JIA. **(a- > c)** Imaging of the right wrist of an 11y old girl with DS. **a** Plain X-ray: no significant bone erosion. **b** Fat suppressed T1-weighted gadolinium-enhanced MRI, frontal view: diffuse mild synovial hypertrophy (arrow heads) with moderate gadolinium enhancement, involving joint synovium (with similar findings on peritendinous synovium). **c** Fat suppressed T2-weighted MRI, axial view: no significant joint or peritendinous effusion. **(d- > f)** Imaging of the right wrist of a 13y old girl with DS **d**. Plain X-Ray: subtle carpal bone erosion (arrow). **e** Fat suppressed T2-weighted MRI, frontal view: bone marrow oedema (*) with no joint effusion. **f**. Fat suppressed T1-weighted gadolinium-enhanced MRI, axial view: diffuse synovial joint enhancement (arrow heads) and bone marrow oedema (*). **(g- > i)** Imaging of the right wrist of a 11y old girl with polyarticular JIA. **g** Plain X-Ray: diffuse severe bone erosion. **h** Fat suppressed T2-weighted MRI, frontal view: joint effusion involving both joint and peritendinous component (*). **i** Fat suppressed T1-weighted gadolinium-enhanced MRI, axial view: diffuse proliferative hypertrophy of joint synovium (arrow heads) with intense gadolinium enhancement

Twelve patients (4 boys, 8 girls) entered the study. Clinical data, imaging findings and laboratory results are summarized in Tables 1 and 2. Imaging findings (MRI and conventional radiography) of DS patients in comparison with findings in RFneg-polyJIA patients are illustrated in Fig. 1.

**Table 1:** Clinical characteristics of patients with Dry Synovitis

Sex	Age at onset (years)	Age at diagnosis (years)	Year of diagnosis	No. joints involved at diagnosis	CRP (mg/l)	ESR (mm/h)	Treatment	Clinical and Imaging findings		
								Imaging at diagnosis	Clinical course	Follow-up imaging
**F**	8	9	2007	18	0,0	12	NSAIDs, CS, MTX, anti-TNF	No synovial thickening, synovial enhancement, joint effusion or joint space narrowing	Good clinical response on anti-TNF treatment, although need for bilateral hip replacement	Progressive destruction of hip joints with joint effusion, joint space narrowing, bone edema and bone erosions
**F**	3	14	2009	30	0,0	8	NSAIDs, CS, MTX, anti-TNF	Diffuse and moderate synovitis on wrists. Bone edema and geodes with discrete joint effusion on hips. Joint space narrowing and erosions on wrists and hips	Persistent, manifest limitation in mobility in multiple joints and need of bilateral hip replacement	Progressive destruction of hip joints with very moderate persistent joint effusion
**F**	4	10,4	2012	31	0,6	8	NSAIDs, CS, MTX	Discrete hips joint effusion and osteochondral involvement with joint space narrowing and erosions	Persistent reduced mobility in the hip	Progressive destruction of hip joints. Development of diffuse and moderate articular and peritendinous synovitis on wrists
**F**	8	9,1	2014	71	5,3	2	NSAIDs, CS, MTX	Diffuse and moderate articular and peritendinous synovitis with no joint effusion	Persistent limitation in mobility of wrists, ankles and elbow	No follow-up imaging available after start of treatment
**M**	1,2	4,9	2013	71	1,4	12	NSAIDs, CS, MTX	Diffuse and moderate articular and peritendinous synovitis with no joint effusion or osteochondral damage	Clinically good response on treatment with persistence of only mild extension deficit in 1 wrist, some PIP joints and the neck	Persistent diffuse and moderate articular and peritendinous synovitis with no destructive damage on cartilage and bone
**M**	16	18,6	2014	26	3,5	7	NSAIDs, CS, MTX, anti-TNF	Diffuse and moderate peritendinous synovitis with osteochondral damage (bone edema and geodes) and no joint effusion.	Persistent reduced mobility in wrists, MCP joints, hip and ankles	No follow-up imaging available after start of treatment
**F**	7	8	2016	71	12,0	15	NSAIDs, CS, MTX, anti-TNF	Diffuse and moderate to intense articular and peritendinous synovitis with very limited bone edema and no joint effusion	Slowly improvement with persistence of mild limitation in mobility of both wrists, PIP 3&4 and MCP 1 & 2 of both hands	No follow-up imaging available after start of treatment
**F**	8	9,7	2017	71	4,1	12	NSAIDs, CS, MTX	Diffuse and moderate to intense articular and peritendinous synovitis with very limited bone edema and no joint effusion	Persistent limitation in the left wrist and ankle	No follow-up imaging available after start of treatment
**F**	2,4	2,9	2012	8	1,6	67	NSAIDs, CS, MTX	Diffuse and moderate articular synovitis	Clinical remission off treatment. Residual joint contractures at multiple joints and osteoporosis	No follow-up imaging available after start of treatment
**F**	5,9	6	2011	22	0,2	6	NSAIDs, CS, MTX	Diffuse and moderate articular and peritendinous synovitis with limited osteochondral damage (bone edema and geodes) and no joint effusion	Persistent reduced mobility of wrists, MCP and PIP joints	Absence of inflammatory signs on MRI
**M**	3,1	5,8	2011	22	0,3	8	NSAIDs, CS, MTX, anti-TNF	Diffuse and moderate articular and peritendinous synovitis with limited bone edema and no joint effusion	Reduced range of motion at wrists, MCP and PIP joints	Osteopenia, joint space narrowing on X-ray
**M**	4,9	10,2	2012	37	0.3	2	NSAIDs, CS, MTX	Diffuse and moderate articular and peritendinous synovitis	Reduced range of motion at both elbows and wrists	Joint space narrowing but no erosions on X-ray

**Table 2:** Comparison between Dry Synovitis and RF-negative polyarticular JIA

Clinical Features		Dry arthritis (no.12)	Poly JIA (no.35)	Significance
**Sex** (female)		8 (66.7%)	33 (94.3%)	**0.03**
**Linear growth**		10 (83.3%)	33 (94.3%)	0.238
**Joints involved at diagnosis** (mean, no.)		32	14	**0.0001**
**Joint involvement**	Upper limbs	12 (100%)	35 (100%)	1.00
	Lower limbs	10 (83.3%)	34 (97.1%)	0.156
	Spine	8 (66.7%)	14 (40%)	0.11
**Symptoms at diagnosis**	Swelling	1 (8.3%)	35 (100%)	**0.0001**
	Joint stiffness	11 (91.7%)	34 (97.1%)	0.45
	Contractures	10 (83.3%)	10 (28.6%)	**0.0001**
	Arthralgia	8 (66.7%)	24 (38.6%)	0.9
**Age at 1**^**st**^** manifestation** (mean, years)		6.1	5.3	0.608
**Age at diagnosis** (mean, years)		8.9	5.8	**0.021**
**Diagnostic delay** (mean, years)		2.9	0.3	**0.0001**
**Follow up duration** (mean, years)		6.7	5.8	0.494

The mean age at first clinical manifestations and at diagnosis of DS were 6.1 years (range 1.3–17) and 8.9 years (2.8–18.5) respectively, displaying a significant diagnostic delay in comparison with polyJIA (2.9 vs 0.3 years, *p* = 0.0001). During the time elapsed between the initial symptoms and final diagnosis of DS, all patients developed functional limitations and/or progressive stiffness. Before consulting a pediatric rheumatology clinic, 7/12 patients were seen by pediatric neurologists or orthopedic surgeons for suspected delayed motor development. Mild pain complaints emerged with time in 8/12 patients.

Clinical examination of DS patients showed symmetric polyarticular joint contractures without clinically palpable synovitis, often associated with a variable degree of muscle atrophy. Both small and large, peripheral and axial joints were involved. None of the DS patients showed clinical signs of systemic inflammation or extra-articular involvement such as uveitis. Follow-up of growth parameters showed a normal linear growth in most patients, according to centiles within parental target height.

The comparison between DS and 35 RF-polyJIA patients is summarized in Table 2. In DS, females were more affected than males, but the female/male ratio was clearly higher in RFneg-polyJIA. The mean age at first clinical manifestations was similar (*p* = 0.608) in both groups, but age at diagnosis was earlier in JIA (5.8 vs 8.9 years, *p* = 0.021). A diagnostic delay of almost 3 years in DS was significantly higher in comparison with RFneg-polyJIA (*p* = 0.0001). The mean number of joints involved at diagnosis, was higher in DS (32 versus 14, *p* = 0.0001) and contractures were significantly more often present (*p* = 0.0001). As expected following the inclusion criteria, joint swelling was absent in the DS cohort. All DS patients were ANA negative while 13/24 (54.1%) polyJIA were ANA positive. RF and anti-CCP antibodies were absent in both DS and polyJIA patients.

Conventional X-rays of hips and/or wrists, available in 10 DS patients at the time of diagnosis, revealed bone erosions in 5 patients who also had osteochondral involvement on MRI (Fig. 1). In comparison with RFneg-polyJIA, the DS group showed a significantly higher frequency of osteopenia (80% vs 13%, *p* = 0.001) and erosions (50% vs 4.3%, *p* = 0.010) during follow-up.

MRI data at diagnosis were available for wrists and/or hips in 10 patients, and for ankle and elbow in one patient each. Abnormalities including synovial, articular and/or osteochondral involvement were found in all patients. Gadolinium-enhanced MRI showed mild synovial inflammation in 9 patients, diffuse moderate synovial thickening in 6, and synovial and tenosynovial enhancement in 9 and 6 cases respectively. Minor joint effusions were seen in three patients only, a peritendinous effusion was found in one case. Bone edema was present in 7 patients and was associated with subchondral geodes in 5. MRI is not usually performed at diagnosis in RFneg-polyJIA. In the present cohort, it was available in only 7 patients (20%). Nevertheless, the picture was significantly different from what observed in DS. All polyJIA patients showed diffuse synovial thickening and enhancement, with clearcut signs of synovial hypertrophy when compared to the DS group. Bone edema was present in only one patient (14.3%), at a frequency significantly lower than in DS (83.3%, *p* = 0.006).

During a mean follow-up of 6.7 years, all DS patients have been treated with NSAIDs, low dose corticosteroids and methotrexate. Five were MTX-resistant, and subsequently received anti-TNF treatment. This treatment approach was similar in the RFneg-polyJIA group. The mean cumulative corticosteroid dose was not significantly different in DS patients (71.6 mg/kg) compared to polyJIA patients (64.3 mg/kg, *p* = 0.44). The response to treatment in DS was fair with only 50% of patients being in clinical remission after MTX treatment for 6 months, compared to 83% of RFneg poly-JIA patients (*p* = 0.04). At the time of the last evaluation, all but one DS patients (pt. no.9) had various degree of disabilities, necessitating joint replacement at the age of young adulthood in two (Table 1). Despite systemic therapy and absence of inflammatory signs on follow-up imaging, most patients showed progressive articular damage with documented osteoporosis in two. Conversely, after a similar treatment approach and comparable follow up of 6 years, 77% of RFneg-polyJIA were in clinical remission and only 23% presented a progressive-erosive arthropathy.

## Discussion

Dry synovitis is a very rare form of arthritis in childhood, which presents discreetly and usually follows a destructive course. Some authors have included this entity within the group of RFneg-polyJIA [4–6] but several characteristics of DS seem to point towards a distinct entity.

In order to properly address this point, we performed a retrospective study on 12 patients with DS seen in a 10-year-time frame (2007–2017) in three tertiary care academic pediatric rheumatology centers.

Our study confirms that progressive, symmetric polyarticular limitations are the core features of DS. What mostly differentiates DS from RFneg-polyJIA are the longer diagnostic delay due to its insidious onset, the higher number of joints affected at diagnosis and the early bone involvement. Pain is rarely mentioned, but mild morning stiffness occurs. Functional limitations in daily life activities are often the predominant complaint at presentation; muscle atrophy may be clinically overt and lead to several non-rheumatological consultations [7]. Laboratory investigations are most often normal, though very slightly elevated levels of acute phase reactants can be observed. MRI with contrast enhancement of affected joints can guide clinicians in the differential diagnosis for patients presenting with progressive articular stiffness without clinical signs of synovitis. MRI showed diffuse synovial thickening and/or enhancement in all but one DS patients. Compared to RFneg-polyJIA, the synovial enhancement was mild with minimal synovial hypertrophy and a less proliferative pattern, sometimes associated with a very limited joint effusion and usually with a diffuse involvement of articular and peritendinous synovium. In patients with a longer follow-up, signs of osteochondral involvement, such as bone edema and geodes, persisted or became evident, despite treatment initiated soon after diagnosis.

When seeing a patient with progressive articular limitations, the diagnosis of DS should be considered in a broad spectrum of differential diagnoses including attenuated forms of type I mucopolysaccharidosis (MPS I), neuromuscular conditions, skeletal dysplasias or chromosomal diseases [8–10].

MPS I is the most common form of mucopolysaccharidosis which results from a deficiency in the lysosomal enzyme α-L-iduronidase [11]. Short stature, characteristic facial appearances and skeletal abnormalities along with history of hernia surgery, frequent respiratory infections, corneal clouding, carpal tunnel syndrome, or cardiac valvular abnormalities must raise a suspicion for MPS. MRI in MPS patients shows thickened cartilage at the hip with no inflammatory nor erosive changes [12].

Neuromuscular diseases, such as collagen type VI-related myopathies, nemaline myopathy and muscular dystrophies, can present with joint contractures [9, 10]. Most of our patients with DS indeed presented with delayed motor development and/or atrophy of the muscular mass but the normal neuromuscular evaluation, muscle enzymes level and MRI helped ruling out a neuromuscular disorder.

Also bone and cartilage dysplasias such as spondyloepiphyseal dysplasia [13], Stickler syndrome [14, 15], camptodactyly arthropathy coxa vara pericarditis syndrome [16] and other collagen-related skeletal dysplasias [17] may present with polyarticular stiffness. The dysmorphic appearance of the patients, a disproportionate short stature and other associated clinical features and characteristic radiographic findings (axial and appendicular abnormalities, epiphysial dysplasia) will guide towards the right diagnosis.

Progressive pseudorheumatoid dysplasia (PPRD) presents with progressive joint stiffness, motor weakness, gait disturbances, joint pain and contractures [18]. Linear growth failure, due to platyspondyly, and radiographs of the hand showing enlarged epiphyses and metaphyses of the metacarpals and phalanges, are the key elements to make the diagnosis.

Among chromosomal diseases, Turner syndrome (TS) can be associated with an inflammatory polyarthropathy which presents in an indolent way, is associated with slightly elevated acute phase reactants as in DS, and is erosive in most of the patients [19]. Again, growth failure, associated characteristic morphological features and the absolute female preponderance provide clues to the correct diagnosis.

Interestingly, Merlin et al. recently published the case of two boys who developed symmetric painless joint contractures preceding by 6 and 12 months the development of superficial plaques of morphea [20]. MRI imaging was similar to what is seen in our DS patients showing absent to moderate thickening of the synovium with mild enhancement after gadolinium infusion. In addition, one of these patients underwent a synovial biopsy which showed dense fibrosis with a sparse inflammatory infiltrate, similar to what was seen in morphea lesions.

Appropriate treatment strategies in DS patients remain a challenge, as documented in our cohort. Indeed, despite treatment with methotrexate and anti-TNF agents resulting in some subjective benefits, polyarticular limitations persisted and joint damage progressed, as documented by imaging. A possible explanation might be related to a different pattern of inflammatory cells and cytokines being involved in DS as compared to RFneg-polyJIA. Of course, the prolonged diagnostic delay may have moved the initiation of treatment to a timepoint where polyarthritis has entered a chronic phase and where osteoarticular damage already occurred. Despite the limited synovial inflammation, DS often follows a destructive course endorsing the need for a better understanding of the disease pathogenesis and the development of alternative effective therapies.

## Conclusion

DS represents a unique entity, clearly distinct from RFneg-polyJIA. Awareness of this rare form of arthropathy by general pediatricians, rheumatologists, neurologists, orthopedic surgeons and geneticists is essential for early recognition and appropriate treatment. Further studies, including synovial pathology, immunology and genetics may contribute to a better understanding of this rare disorder.

## Data Availability

The datasets generated and/or analysed during the current study are not publicly available (contains patients information).

## Abbreviations

ANA: Antinuclear antibodies
CRP: C-reactive protein
DS: Dry synovitis
ESR: Erythrocyte sedimentation rate
F: Female
JIA: Juvenile idiopathic arthritis
M: Male
polyJIA: Polyarticular JIA
RF: Rheumatoid Factor
RFneg-polyJIA: Polyarticular juvenile idiopathic arthritis
